# Outpatient discharge management model: a strategy to promote continuity of care

**DOI:** 10.1590/0034-7167-2025-0221

**Published:** 2026-03-30

**Authors:** Daniele Cristina dos Reis Bobrowec, Elizabeth Bernardino, Gisele Knop Aued, Jaqueline Dias do Nascimento Selleti, Maria Manuela Ferreira Pereira da Silva Martins, Camila Rorato, Keyla Cristina da Rocha Fracarolli, Lívia Cozer Montenegro

**Affiliations:** I Universidade Federal do Paraná. Curitiba, Paraná, Brazil; II Universidade Federal do Rio Grande do Sul. Porto Alegre, Rio Grande do Sul, Brazil; III Universidade do Porto. Porto, Portugal

**Keywords:** Ambulatory Care, Patient Discharge, Continuity of Patient Care, Healthcare Models, Nurse’s Role., Atención Ambulatoria, Alta del Paciente, Continuidad de la Atención al Paciente, Modelos de Atención de Salud, Rol de la Enfermera.

## Abstract

**Objectives:**

to propose an outpatient discharge management model from specialized care to primary care to promote continuity of care for chronic conditions.

**Methods:**

action research with a qualitative approach. Twenty-eight professionals, including physicians, nurses, and public administrators, participated. Data collection was carried out through participant observation, documentary research, semi-structured interviews, and workshops. The data were subjected to Creswell’s content analysis.

**Results:**

eight categories and 22 subcategories supported the creation of the outpatient discharge management model, emphasizing communication among levels of the healthcare network, designating the nurse as the process coordinator, and encouraging collaborative practices.

**Final Considerations:**

the study proposed an outpatient discharge management model focused on continuity of care, in addition to reinforcing the role of nurses, highlighting attention to communication, technological support and collaborative practices, reaffirming the importance of Primary Health Care as a care organizer.

## RESUMEN

**Objetivos:**

proponer un modelo de gestión para el alta ambulatoria de atención especializada a atención primaria, con el fin de promover la continuidad de la atención a las enfermedades crónicas.

**Métodos:**

investigación-acción con enfoque cualitativo. Participaron veintiocho profesionales, entre ellos médicos, enfermeros y administradores públicos. La recopilación de datos se realizó mediante observación participante, investigación documental, entrevistas semiestructuradas y talleres. Los datos se sometieron al análisis de contenido de Creswell.

**Resultados:**

ocho categorías y 22 subcategorías respaldaron la creación del modelo de gestión del alta ambulatoria, priorizando la comunicación entre los niveles de la red de atención, designando a la enfermera como coordinadora del proceso y fomentando las prácticas colaborativas.

**Consideraciones Finales:**

el estudio propuso un modelo de gestión del alta ambulatoria centrado en la continuidad de la atención, además de reforzar el papel del enfermero, destacando la atención a la comunicación, el apoyo tecnológico y las prácticas colaborativas, reafirmando la importancia de la Atención Primaria de Salud como organizadora de la atención.

## INTRODUCTION

The aging of the world’s population, resulting from increased life expectancy and reduced mortality rates from infectious diseases, has favored the emergence of chronic health conditions, a major challenge for health systems^(1)^.

People with chronic health conditions have multiple comorbidities that last for a long time and, in some cases, are permanent. They are dependent on the system and require continuous and comprehensive care from different professionals^(2-4)^.

The Brazilian Health System (In Portuguese, *Sistema Único de Saúde* - SUS) is organized into a Healthcare network (In Portuguese, *Rede de Atenção à Saúde* - RAS), a structure comprised of various healthcare services committed to providing continuous and comprehensive care to a specific population. For care to be delivered efficiently, coordination among levels of care is essential, and this is achieved through healthcare regulation^(2,3)^. This process involves both referral, characterized by referral of patients from Primary Health Care (PHC) to more specialized levels, and counter-referral, which consists of patients returning to PHC after the problem has been resolved or the health condition has been stabilized^(6-8)^.

However, despite counter-referral being provided for in SUS regulations, its implementation faces several barriers. Notable challenges include insufficient network structure, poor communication among services, professionals’ lack of knowledge about the RAS’s functioning, and the PHC’s weakness in absorbing more complex cases. These difficulties contribute to prolonged patient stays in highly complex services, which overloads tertiary hospitals and exacerbates problems such as waiting lists for specialist appointments, tests, and surgical procedures^(9-11)^.

In the context of teaching hospitals, which play an essential role in tertiary care, counter-referral becomes even more challenging. Patients referred to these services often remain involved for long periods, even when they could already be treated in primary care or at intermediate levels of care. The lack of structured secondary care and insufficient resources in primary care hinder the transition of these patients and, consequently, perpetuate their dependence on the tertiary level^(12-14)^.

As a result of this inadequate management of care flows, we observed a scenario of overcrowded hospital emergency rooms, long waiting times, and limited access for new patients who truly require specialized care. This situation is especially worrying for chronic patients, who require continuous and individualized monitoring to maintain their quality of life^(2,3,15)^.

In this context, several strategies have been implemented to improve healthcare regulation and optimize flows within the RAS. Initiatives such as PHC expansion, introduction of programs as “*Melhor em Casa*” (Better at Home) and Home Care Service, and remote patient monitoring have been adopted to reduce pressure on hospital services^(16-18)^. However, additional challenges arise, such as the impact of emerging diseases (COVID-19, dengue, avian flu), which further burden the health system and make it difficult to organize the RAS^(19,20)^.

In this scenario, outpatient discharge management can be seen as one alternative to reduce the demand for emergency services, promote continuity of care, and optimize the use of available resources. Implementing a structured outpatient discharge model could help identify patients who truly need to remain in tertiary care, who can be monitored in PHC with adequate support, and who require intermediate care. To achieve this, coordinated planning is essential to ensure continuity of care, reducing excessive reliance on specialized services.

Based on this perspective, the *Complexo do Hospital de Clínicas da Universidade Federal do Paraná* (CHC-UFPR) implemented an outpatient discharge management service in 2023. This service is based on the same principles as the inpatient discharge management service established at the institution in 2018^(21)^. This service consists of establishing guidelines for the safe and effective transition of patients, particularly transition from specialized outpatient care to PHC. Thus, considering the institution’s experience with discharge management, the context outlined, and the need to develop a scientifically based outpatient discharge management model, the following guiding question arises: how can an outpatient discharge management model be developed to promote continuity of care for people with chronic conditions?

## OBJECTIVES

To propose an outpatient discharge management model from specialized care to primary care to promote continuity of care for chronic conditions.

## METHODS

### Ethical aspects

This study complied with the ethical principles set forth in Resolution 466/12 of the Brazilian National Health Council. This research was conducted with the approval of a Research Ethics Committee. Data collection began only after participants’ acceptance, expressed by reading and signing the Informed Consent Form. To ensure participant anonymity, each participant was assigned an identifying code.

### Study design

This is qualitative action research study, according to the Equator Network’s COnsolidated criteria for REporting Qualitative research guidelines^(22)^.

### Study setting

The research was carried out at CHC-UFPR, and included the care management division areas, contracting and regulation sector, and outpatient unit focused on specialized outpatient care.

### Data source

The data sources used for this study were four:

1. Participant observation in the outpatient discharge management service;
2. Documentary research in outpatient discharge management service records;
3. Semi-structured interview with physicians and nurses who worked in the institution’s outpatient care, chosen by convenience;
4. Workshops with professionals working in specialized outpatient care, discharge management and care management, selected by convenience.

### Data collection

Data collection took place from May 29 to December 15, 2023, and was conducted by one of the study authors. For participant observation and documentary research, the researcher contacted the head of the outpatient discharge management service directly to request authorization and determine the appropriate period for data collection.

Participant observation took place in the outpatient discharge management service room, monitoring professionals’ activities in this service; it lasted 12 hours from May 29 to June 2, 2023.

In the documentary research, the following documents from the outpatient discharge management service were assessed: Project Model Canvas for implementation; history of this service; documents used in routine (standard operating procedures, flows, forms, reports); and field diary, for a total of 36 hours, from June 3 to June 10, 2023.

The semi-structured interview consisted of questions to characterize participants, including six open-ended questions. The instrument was pilot-tested to assess its feasibility, estimated interview time, and whether it would meet the research objective. Fourteen physicians and 12 nurses were invited to participate in the interviews, with eight physicians and nine nurses agreeing to participate. Interviews lasted an average of ten minutes from June 29 to September 28, 2023. Data collection was concluded based on theoretical data saturation.

Six workshops were held to consolidate the findings for constructing an outpatient discharge management model and develop the necessary tools for its application in healthcare practice. Two physicians, 14 nurses, and two public administrators were invited, with ten nurses and one public administrator participating in the workshops from November 24 to December 15, 2023. Each workshop lasted, on average, 1 hour and 30 minutes.

All participants were selected by convenience, from a total of 28 professionals, including nurses, physicians, and public administrators working at the institution. They were recruited by verbal invitation or virtual invitation via WhatsApp^®^ text message. Interviews were scheduled based on each participant’s availability, and workshop days and times were chosen to accommodate the majority of professionals invited to participate.

### Data analysis

Data analysis was conducted through content analysis, as proposed by Creswell^(23)^. All documents, transcripts, observation results, interviews and workshops were identified, classified and entered into Atlas.ti^®^ software, totaling 35 documents organized by origin and type.

Subsequently, a careful reading of the data was performed to immerse oneself in the information and understand the general meaning of the analyzed material. The data were then coded through a detailed rereading, assigning codes to excerpts, using Atlas.ti^®^ software to support the identification and classification of implicit meanings. This coding enabled the definition of topics, resulting in the identification of eight main categories and 22 subcategories. Finally, data interpretation enabled the development of an outpatient discharge management model, as well as the identification of those involved and the factors influencing the process.

## RESULTS

The survey participants were 28 professionals from the institution, of which 67% were nurses, 29% were physicians, and 4% were public management technologists. Seventy-four percent of participants were female, and 26% were male. Concerning age, 46% were between 30 and 40 years old, 35% were between 41 and 50 years old, and 14% were over 50 years old. The majority (75%) had worked at the institution for more than five years.

Participant observation allowed the researcher to understand the outpatient discharge management process at CHC-UFPR and understand the established routines and workflows. While the observation provided insight into the service’s activities, documentary research supplemented the information about the service’s routines, standards, and workflows.

Professionals’ perspectives shed light on this issue through interviews that detailed the context under study. Furthermore, it was possible to highlight the contributions of those who experience outpatient care daily to improving processes related to the discharge of people with chronic health conditions.

The workshops provided an opportunity to discuss existing workflows with a group of professionals from areas related to outpatient care, revealing areas for improvement to be implemented.

Finally, data from all stages contributed to the composition of categories related to the outpatient discharge process, which demonstrate potential strategies for success, challenges, needs, and stakeholders, from the perspective of specialized care professionals. Chart 1 presents the categories and subcategories found in data analysis.

**Chart 1:** Categories and subcategories related to outpatient discharge management, Curitiba, Paraná, Brazil

CATEGORY	SUBCATEGORY
Interpersonal and interprofessional communication	Communication with patients
	Communication among hospital teams
	Communication among the different levels of the Healthcare Network
Weakness of knowledge for adequate outpatient discharge	Lack of clinical knowledge among Primary Health Care professionals
	Lack of knowledge about the resources of the healthcare network
	(Lack) of knowledge about the counter-referral process
Criteria for outpatient discharge	Establishing criteria for outpatient discharge
	People with potential for outpatient discharge
	People who require specialized care support for continuity of care in Primary Health Care
Difficulties in counter-referring people from the outpatient clinic to Primary Health Care	Culture
	Weaknesses in the healthcare network organization
	Personal insecurity
	Professional insecurity
Resources for adequate counter-referral	Required professionals
	Standardized processes for counter-referral
	Relevant tools/support for process safety
	Use of health information and communication technologies
Nurses’ role in outpatient discharge management	Education for outpatient discharge
	Coordination of outpatient discharge
Collaborative practices	Safety for promoting continuity of care in Primary Health Care
	Teamwork
	Joint protocols
Co-responsibility of a person and/or family member for self-care	

From the categories found, it is possible to highlight that “Interpersonal and interprofessional communication”, “Weakness of knowledge for adequate outpatient discharge”, “Difficulties in counter-referring people from the outpatient clinic to Primary Health Care” and “Resources for adequate counter-referral” present the great challenges experienced by the institution and the RAS to consolidate continuity of care for people with chronic diseases.

*I think communication is one of the main factors why I am unable to refer this patient to the UBS.* (Profess. R)

*I don’t know if the processes are clear either, because I’d have to fill them out, but I never access them. How do you make a counter-referral? I’ve done a few, but you have to fill out some document, I don’t know where. I don’t know if this process is completely clear to us, I guess.* (Profess. N)

*Maybe if we had an integrated system, right? Because here we give patients a piece of paper. Whether that paper will actually get there, physically, I don’t know.* [...] *and really, the non-unified electronic digital systems, I think that’s something that makes it difficult.* (Profess. O)

In turn, the “Criteria for outpatient discharge”, “Nurses’ role in outpatient discharge management”, “Collaborative practices” and “Co-responsibility of a person and/or family member for self-care” categories highlight possible strategies for achieving this continuity.

*Most of the time, it goes like this: we evaluate patients; we see who has less serious illnesses and who has all the medications and tests available to continue at the health unit; we make a counter-referral; and discharge them from the outpatient clinic.* (Profess. M)

[...] *we can provide patients with all the necessary information again. We inform them that they are going to the primary care unit, that they will be monitored, that everything is stipulated and that the physician can monitor them* [...] *so, we explain all of this to them during this nursing consultation*. (Profess. W)

[...] *now, what is each person’s responsibility? One is ours, the other is primary care, and patients has his own responsibility for his own treatment.* [...] *ours ended here; primary care begins there; and here the patient’s begins.* (Profess. A)

**Figure 1:**
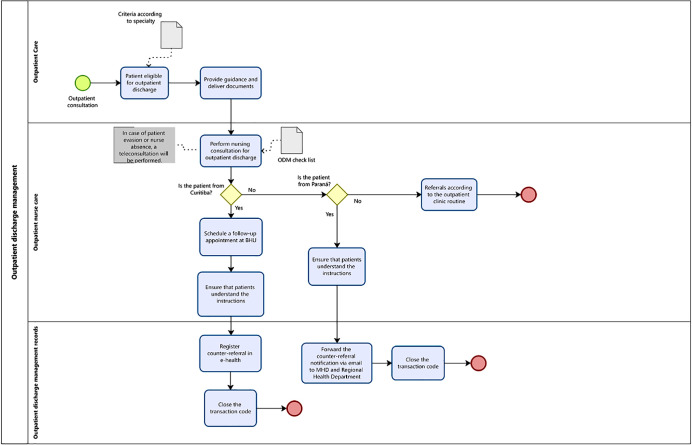
Outpatient discharge management process flow, Curitiba, Paraná, Brazil

As a result of the workshops, the outpatient discharge management process flow was developed and validated by study participants in the final workshop. This process occurs at three specific stages: specialty care; outpatient nurse care; and outpatient discharge management record (Figure 1).

*ODM - outpatient discharge management; BHU - Basic Health Unit; MHD - Municipal Health Department.*

**Figure 2:**
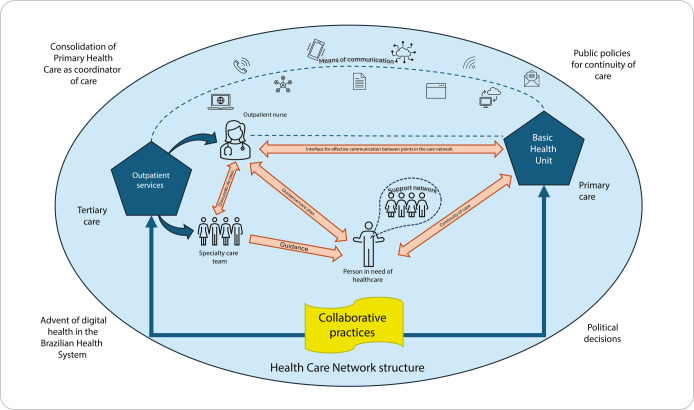
Outpatient discharge management model, Curitiba, Paraná, Brazil

The analysis of all the data collected in the study provided the researcher with a deep understanding of the context under study and provided the foundation for developing the outpatient discharge management model^(24)^. Figure 2 represents the idealized outpatient discharge management model.

The creation of this model places the person in need of healthcare at the center of the process, focusing on ensuring their smooth transition from tertiary care outpatient services to the referral Basic Health Unit, promoting continuity of care. It is important to emphasize that healthcare professionals, both in primary and tertiary care, must work alongside the person, and the person, in turn, must be an active agent in the care process.

Communication is essential for the proper functioning of the process, both between professionals, individuals and their family, and among professionals themselves, within the institution or among the different levels of the RAS. Creating an effective communication interface among the tertiary and primary levels is crucial and depends on regulatory tools, communication channels, and organizational agreements, which, in the proposed model, are represented by a dotted line connecting both levels. This dotted line symbolizes the constant improvement of communication, adapting to technological and social changes, and the development of SUS organizational arrangements, especially with regard to RAS regulation.

Designating a clinical nurse as a central link among the RAS levels strengthens continuity of care and highlights the importance of this professional in outpatient care. Collaboration among all involved is essential to achieving the discharge process objectives, both in relationships among professionals and among patients and their support network, and among the different levels of the RAS. However, this collaboration depends almost exclusively on professionals’ commitment, and it is up to managers to encourage and provide a favorable environment for the development of collaborative practices throughout the process.

The proposed model was developed based on the reality of a tertiary teaching hospital. It can be inferred that, for its implementation, the step-by-step process to be followed should consider the following stages:

- Define patients flow in the RAS and in the institution, alignment of referral and counter-referral;
- Identify critical nodes of the institution, patient profile, number of linked patients, outpatient care capacity and its impact on the dynamics of emergency care and hospital admission/readmission rates;
- Define outpatient discharge criteria, which must consider patients’ conditions for discharge from specialized care and primary care’s ability to offer the necessary support for continuity of care;
- Establish an effective communication flow between the institution’s care team and care regulation with the network teams;
- Involve patients and/or family members throughout the entire process, from referral to specialized care, clarifying the pathways within the care network, the comings and goings so that they do not feel abandoned at the time of counter-referral;
- Establish standardized actions with the local manager to facilitate scheduling follow-up appointments in primary care and completing the transaction code, as agreed with the institution.

Carrying out a pilot project with a specialty with a smaller number of patients may be an acceptable choice for aligning weak points in the initial process.

For this model to be viable on a large scale, it needs to be incorporated into public policies that consolidate care processes within the SUS, respecting the principles of universality, comprehensiveness, and equity. The operationalization of the model requires clear outpatient discharge criteria by specialty, nurses’ leading role in the discharge management process, patients’ commitment to their own health, the network’s collaboration to ensure that follow-up appointments at the Basic Health Unit are scheduled by a specialized care nurse, and encouragement of collaborative practices at all levels of care.

## DISCUSSION

Content analysis revealed important aspects for developing the idealized model. Among these, interprofessional and interpersonal communication stands out, emerging as a key factor in managing outpatient discharge. This communication is crucial both among healthcare professionals and individuals, as well as among the institution’s own professionals and professionals at different levels of the RAS. This process involves the exchange of information relevant to patient care, considering the past, present, and future plans. Poor communication between healthcare professionals and individuals/families can compromise continuity of care^(3,8,25,26)^.

Efficient continuity of care is directly related to interactions among different stakeholders throughout the care pathway, adequate communication, and a person-centered interprofessional approach. Failures in the exchange of interprofessional information can result in duplicate appointments or unnecessary tests, while failures in providing guidance to patients under care can lead to poor treatment adherence and, consequently, inappropriate medication use and a lack of understanding of their own role in self-care^(4,25,27)^.

Another important aspect is that professionals involved in outpatient discharges must be well-informed about RAS resources and counter-referral processes. Professionals who master these procedures are familiar with the services available in the network and understand patients’ individual needs, making them better prepared to ensure transition of care after outpatient discharge^(3)^.

Defining criteria for outpatient discharge can help identify individuals eligible for discharge as well as support professionals in developing joint strategies with PHC to ensure continuity of care for patients with complex conditions. Difficulties in counter-referring individuals from specialized outpatient clinics to PHC demonstrate the RAS’s fragility, especially regarding access to healthcare services. Facilitating this access is essential to promote the much-desired continuity, allowing people to receive care for their health condition according to their needs^(12)^. The barrier to access reinforces patients’ and professionals’ insecurity about the quality of PHC, which increases resistance to outpatient discharge^(2,3)^.

To enable effective counter-referral, it is essential to have qualified professionals committed to network collaboration. Studies show that liaison nurses play a fundamental role in the transition and continuity of care between hospital services and outpatient care^(28-31)^. Furthermore, well-structured processes and integrated computerized systems are essential to optimize outpatient discharge management^(32-36)^.

Nurses’ role in coordinating outpatient discharge is essential, especially through educational initiatives. The proposed outpatient discharge management model seeks to reestablish the bond between patients and PHC, promoting open and effective communication, the sharing of relevant information, and engagement in decision-making regarding care. This is not limited to clinical information transmission, but also involves strengthening the relationship of trust and mutual respect among all involved^(35,36)^. Health education is essential for enabling patients and family members to understand their treatments and how to access services available through the RAS. This educational process, in which nurses play a central role, is essential for patients to take an active role in their own care^(35-37)^.

The adoption of collaborative practices among the various stakeholders of the RAS will contribute to the effectiveness of continuity of care and the consolidation of SUS principles. To this end, it is essential that professionals are committed to promoting continuity of care^(38)^. Furthermore, it is essential to structure and standardize the exchange of information among services^(39,40)^.

Responsible participation by individuals and/or family members is a determining factor for continuity of care. For this to occur, they must understand the RAS services and know how to access them^(25)^. Therefore, an integrated, person-centered approach, based on trust, effective communication, and interprofessional collaboration, is essential to promote a safe transition and quality care throughout the health system.

### Study limitations

Limitations include the limited research on the topic and the lack of full consolidation of the proposed model, which still requires future research to assess its impact. The challenge lies in definitively structuring the model and mobilizing managers to expand it, an aspect that will be explored in future studies.

### Contributions to nursing, health or public policy

This study contributes significantly to the field of nursing by highlighting the central role of nurses in managing the outpatient discharge process and ensuring continuity of care. Furthermore, it demonstrates the need to implement public policies to ensure continuity of care and consolidate PHC as the coordinator of healthcare within the SUS.

## FINAL CONSIDERATIONS

This research enabled the development of a proposed care model for outpatient discharge management, emphasizing continuity of care and nurses’ role as coordinator of this process. It was evident that the model’s effectiveness depends on clear and efficient communication, adequate information sharing, and acknowledgment of nurses as a liaison. Additionally, training professionals on the resources available in the network, combined with the creation of well-defined protocols built in an integrated manner between specialized care and PHC, can strengthen collaboration among the different levels of the RAS. However, the success of this strategy depends, fundamentally, on patients’ engagement as leading actors of their own care.

## Data Availability

The research data are available only upon request.
