# Supplementary material for: Outpatient discharge management model: a strategy to promote continuity of care
Source: Rev Bras Enferm. 2026 Mar 30;79:e20250221. doi: 10.1590/0034-7167-2025-0221 (PMC13035287; doi:10.1590/0034-7167-2025-0221)
Supplement: Translation

# Translation: Modelo de gestão de alta ambulatorial: estratégia para promover a continuidade do cuidado

**DOI:** 10.1590/0034-7167-2025-0221pt

**Authors:** Daniele Cristina dos Reis Bobrowec, Elizabeth Bernardino, Gisele Knop Aued, Jaqueline Dias do Nascimento Selleti, Maria Manuela Ferreira Pereira da Silva Martins, Camila Rorato, Keyla Cristina da Rocha Fracarolli, Lívia Cozer Montenegro

**Affiliations:** I Universidade Federal do Paraná. Curitiba, Paraná, Brasil; II Universidade Federal do Rio Grande do Sul. Porto Alegre, Rio Grande do Sul, Brasil; III Universidade do Porto. Porto, Portugal

**Keywords:** Assistência Ambulatorial, Alta do Paciente, Continuidade da Assistência ao Paciente, Modelos de Assistência à, Saúde, Papel do Profissional de Enfermagem.

## Abstract

**Objetivos:**

propor modelo de gestão de alta ambulatorial da atenção especializada para a atenção primária para promover a continuidade do cuidado nas condições crônicas.

**Métodos:**

pesquisa-ação, com abordagem qualitativa. Participaram 28 profissionais, como médicos, enfermeiros e gestores públicos. Coleta de dados ocorreu pela observação participante, pesquisa documental, entrevistas semiestruturadas e oficinas. Os dados foram submetidos à análise de conteúdo proposta por Creswell.

**Resultados:**

oito categorias e 22 subcategorias ampararam a criação do modelo de gestão de alta ambulatorial, enfatizando a comunicação entre os níveis da Rede de Atenção à Saúde, designando o enfermeiro como coordenador do processo e incentivando práticas colaborativas.

**Considerações Finais:**

o estudo propôs um modelo de gestão de alta ambulatorial voltado para a continuidade do cuidado, além de reforçar o papel do enfermeiro, destacar a atenção para a comunicação, suporte tecnológico e práticas colaborativas, reafirmando a importância da Atenção Primária à Saúde como ordenadora do cuidado.

## INTRODUÇÃO

O envelhecimento da população mundial decorrente do aumento na expectativa de vida e da redução das taxas de mortalidade por doenças infectocontagiosas favoreceu o advento das condições crônicas de saúde, um grande desafio a ser enfrentado pelos sistemas de saúde^(1)^.

Pessoas com condições crônicas de saúde possuem múltiplas comorbidades, de curso prolongado e, em alguns casos, de forma permanente. São dependentes do sistema e necessitam de atendimento contínuo e integral por diferentes profissionais^(2-4)^.

No Brasil, o Sistema Único de Saúde (SUS) está organizado em Rede de Atenção à Saúde (RAS), que é estrutura composta por diversos serviços de saúde, comprometida em fornecer assistência contínua e integral a uma determinada população. Para que o cuidado ocorra de maneira eficiente, a articulação entre os níveis de atenção é fundamental, sendo realizada por meio da regulação assistencial^(2,3)^. Esse processo envolve tanto a referência, caracterizada pelo encaminhamento de pacientes da Atenção Primária à Saúde (APS) para níveis mais especializados, quanto a contrarreferência, que consiste no retorno do paciente para a APS após a resolução do problema ou estabilização da condição de saúde^(6-8)^.

Entretanto, apesar de a contrarreferência estar prevista nas normativas do SUS, sua implementação enfrenta diversas barreiras. Entre os desafios, destacam-se a estruturação insuficiente da rede, a comunicação deficiente entre os serviços, o desconhecimento dos profissionais sobre o funcionamento da RAS e a fragilidade da APS na absorção de casos de maior complexidade. Essas dificuldades contribuem para a permanência prolongada de paciente em serviços de alta complexidade, o que sobrecarrega hospitais terciários e agrava problemas como filas de espera para consultas especializadas, exames e procedimentos cirúrgicos^(9-11)^.

No contexto dos hospitais de ensino, que possuem papel essencial na atenção terciária, a contrarreferência se torna ainda mais desafiadora. Pacientes encaminhados para esses serviços frequentemente permanecem vinculados por longos períodos, mesmo quando já poderiam ser acompanhados na APS ou em níveis intermediários de atenção. A ausência de uma atenção secundária estruturada e a insuficiência de recursos na APS dificultam a transição desses pacientes e, por conseguinte, perpetuam a dependência do nível terciário^(12-14)^.

Como resultado dessa inadequada gestão dos fluxos assistenciais, observa-se um cenário de emergências hospitalares superlotadas, filas prolongadas e limitação de acesso para novos pacientes que realmente necessitam de atenção especializada. Essa situação é especialmente preocupante para pacientes crônicos, que demandam acompanhamento contínuo e individualizado para manutenção da qualidade de vida^(2,3,15)^.

Diante desse contexto, algumas estratégias vêm sendo implementadas para aprimorar a regulação assistencial e otimizar os fluxos dentro da RAS. Iniciativas como a expansão da APS, a introdução de programas como “Melhor em Casa” e Serviço de Atenção Domiciliar, e o monitoramento remoto de pacientes têm sido adotadas com o intuito de reduzir a pressão sobre os serviços hospitalares^(16-18)^. No entanto, desafios adicionais surgem, como o impacto de doenças emergentes (COVID-19, dengue, gripe aviária), as quais sobrecarregam ainda mais o sistema de saúde e dificultam a organização da RAS^(19,20)^.

Nesse cenário, a gestão de alta ambulatorial pode ser vista como uma das alternativas para tentar reduzir a procura aos serviços de urgência/emergência, promover a continuidade do cuidado e otimizar a utilização dos recursos disponíveis. A implementação de um modelo estruturado de alta ambulatorial poderia auxiliar na identificação dos pacientes que realmente necessitam permanecer vinculados ao nível terciário, diferenciando dos que podem ser acompanhados na APS com suporte adequado e dos que necessitam de atenção intermediária. Para isso, é fundamental um planejamento articulado que assegure a continuidade dos cuidados, de modo a reduzir a dependência excessiva dos serviços especializados.

Com base nessa perspectiva, o Complexo do Hospital de Clínicas da Universidade Federal do Paraná (CHC-UFPR) implantou, em 2023, o serviço de gestão de alta ambulatorial, pautado no mesmo fundamento do serviço de gestão de alta para pacientes internados existente na instituição desde 2018^(21)^, que consiste em estabelecer diretrizes para transição segura e eficaz dos pacientes, com a particularidade da transição do cuidado do ambulatório especializado para a APS. Assim, considerando a experiência adquirida na instituição acerca da gestão da alta, o contexto delineado e a necessidade de elaborar um modelo de gestão de alta ambulatorial pautado em um método científico, tem-se a seguinte questão norteadora: como elaborar um modelo de gestão de alta ambulatorial para promoção da continuidade do cuidado de pessoas com condições crônicas?

## OBJETIVOS

Propor modelo de gestão de alta ambulatorial da atenção especializada para a atenção primária para promover a continuidade do cuidado de pessoas com condições crônicas de saúde.

## MÉTODOS

### Aspectos éticos

Para a realização deste estudo, foram considerados os preceitos éticos previstos na Resolução nº 466/12 do Conselho Nacional de Saúde do Brasil. Esta pesquisa foi realizada mediante a aprovação do Comitê de Ética em Pesquisa. A coleta de dados iniciou somente após aceite dos participantes, expresso por meio da leitura e assinatura do Termo de Consentimento Livre e Esclarecido. Para garantir o anonimato dos participantes, foi atribuído um código identificador a cada um.

### Tipo de estudo

Estudo de abordagem qualitativa do tipo pesquisa-ação, conforme as diretrizes do *COnsolidated criteria for REporting Qualitative research* da Rede Equator^(22)^.

### Cenário do estudo

A pesquisa foi realizada no CHC-UFPR, e compreendeu as áreas da divisão de gestão de cuidado, setor de contratualização e regulação e unidade de ambulatórios voltadas à assistência ambulatorial especializada.

### Fonte de dados

As fontes de dados utilizadas para o presente estudo foram quatro:

1. Observação participante no serviço de gestão de alta ambulatorial;
2. Pesquisa documental em registros do serviço de gestão de alta ambulatorial;
3. Entrevista semiestruturada com médicos e enfermeiros que atuavam na assistência ambulatorial da instituição, escolhidos por conveniência;
4. Oficinas com profissionais atuantes na atenção ambulatorial especializada, gestão de altas e gestão assistencial, selecionados por conveniência.

### Coleta de dados

A coleta de dados ocorreu de 29 de maio a 15 de dezembro de 2023, realizada por uma das autoras do estudo. Para a observação participante e pesquisa documental, a pesquisadora estabeleceu contato direto com a chefia do serviço de gestão de alta ambulatorial para solicitar autorização e definir o período adequado para a coleta de dados.

A observação participante ocorreu na sala do serviço de gestão de alta ambulatorial, acompanhando as atividades das profissionais deste serviço; foram 12 horas no período de 29 de maio a dois de junho de 2023.

Na pesquisa documental, foram avaliados os seguintes documentos do serviço de gestão de alta ambulatorial: *Project Model Canvas* para implantação; histórico desse serviço; documentos utilizados na rotina (procedimentos operacionais padrão, fluxos, formulários, relatórios); e diário de campo, em um total de 36 horas, de 03 de junho a 10 de junho de 2023.

A entrevista semiestruturada foi composta por questões para caracterização dos participantes, incluindo seis perguntas abertas. O instrumento utilizado foi submetido a teste piloto para avaliar sua viabilidade de aplicação, tempo estimado da entrevista e se atenderia ao objetivo de pesquisa. Foram convidados para as entrevistas 14 médicos e 12 enfermeiros, dos quais aceitaram participar oito médicos e nove enfermeiros, no período de 29 de junho a 28 de setembro de 2023, com média de duração de dez minutos para cada entrevista. A coleta de dados foi encerrada pelo critério de saturação teórica dos dados.

Foram realizadas seis oficinas para consolidar os achados para construção do modelo de gestão de alta ambulatorial e desenvolver os instrumentos necessários para aplicá-lo na prática assistencial. Foram convidados dois médicos, 14 enfermeiros e dois gestores públicos, dos quais participaram das oficinas dez enfermeiros e um gestor público no período de 24 de novembro a 15 de dezembro de 2023. Cada oficina teve, em média, 1 hora e 30 minutos de duração.

Todos os participantes foram selecionados por conveniência, em um total de 28 profissionais, entre entrevistas e oficinas, sendo enfermeiros, médicos e gestores públicos atuantes na instituição. Estes foram recrutados por convite verbal ou virtual, por mensagem de texto via *WhatsApp* ^®^. As entrevistas foram agendadas conforme a disponibilidade de cada participante e, para o agendamento das oficinas, foram definidos dias e horários que viabilizassem a participação da maioria dos profissionais convidados a participar do estudo.

### Análise dos dados

A análise dos dados foi conduzida por meio da análise de conteúdo, conforme proposta de Creswell^(23)^. Todos os documentos, transcrições, resultados de observações, entrevistas e oficinas foram identificados, classificados e inseridos no *software* Atlas.ti^®^, totalizando 35 documentos organizados por origem e tipo.

Posteriormente, realizou-se leitura criteriosa dos dados para imersão nas informações e compreensão do sentido geral do material analisado. Em seguida, os dados foram codificados por meio de uma releitura detalhada, com atribuição de códigos aos trechos, utilizando o *software* Atlas.ti^®^ como apoio na identificação e classificação dos significados implícitos. Essa codificação possibilitou a definição dos temas, resultando na identificação de oito categorias principais e 22 subcategorias. Por fim, a interpretação dos dados permitiu a elaboração de um modelo de gestão de alta ambulatorial, bem como identificar os envolvidos e os fatores influentes no processo.

## RESULTADOS

Os participantes da pesquisa foram 28 profissionais da instituição, dos quais 67% eram enfermeiros, 29%, médicos, e 4%, tecnólogos em gestão pública. Eram do sexo feminino 74% dos participantes, e do sexo masculino, 26%. Em relação à faixa etária, 46% tinham entre 30 e 40 anos de idade, 35%, entre 41 e 50 anos, e 14%, mais de 50 anos de idade. A maior parte (75%) atuava na instituição há mais de cinco anos.

A observação participante possibilitou à pesquisadora conhecer o processo de gestão de altas ambulatoriais no CHC-UFPR, compreender as rotinas e os fluxos estabelecidos. Entende-se que, se as horas de observação permitiram vivenciar as atividades desse serviço, a pesquisa documental complementou as informações acerca das rotinas, normas e fluxos do processo de trabalho do serviço.

A visão dos profissionais trouxe luz a esta problemática por meio de entrevistas que detalharam a realidade estudada. Além disso, foi possível evidenciar as contribuições daqueles que vivenciam a assistência ambulatorial diariamente para melhoria dos processos relacionados à alta ambulatorial de pessoas com condições crônicas de saúde.

As oficinas oportunizaram a discussão dos fluxos existentes com um grupo de profissionais de áreas relacionadas à assistência ambulatorial, desvelando pontos de melhoria a serem implementados.

Por fim, os dados de todas as etapas contribuíram para composição das categorias referentes ao processo de alta ambulatorial, as quais demonstram estratégias potenciais para o sucesso, os desafios, as necessidades e os atores envolvidos, pela perspectiva dos profissionais da atenção especializada. O Quadro 1 apresenta as categorias e subcategorias encontradas na análise dos dados.

**Quadro 1:** Categorias e subcategorias relacionadas à gestão de alta ambulatorial, Curitiba, Paraná, Brasil

CATEGORIA	SUBCATEGORIA
Comunicação interpessoal e interprofissional	Comunicação com a pessoa em acompanhamento
	Comunicação entre as equipes do hospital
	Comunicação entre os diferentes níveis da Rede de Atenção à Saúde
Fragilidade de conhecimento para uma adequada alta ambulatorial	Carência de conhecimento clínico dos profissionais da Atenção Primária à Saúde
	Falta de conhecimento dos recursos da Rede de Atenção à Saúde
	(Des)Conhecimento do processo de contrarreferência
Critérios para alta ambulatorial	Estabelecimento de critérios para a alta ambulatorial
	Pessoas com potencialidade para alta ambulatorial
	Pessoas que necessitam de suporte da atenção especializada para a continuidade do cuidado na Atenção Primária à Saúde
Dificuldades para a contrarreferência da pessoa do ambulatório para a Atenção Primária à Saúde	Cultura
	Fragilidades na organização da Rede de Atenção à Saúde
	Insegurança da pessoa
	Insegurança do profissional
Recursos para uma adequada contrarreferência	Profissionais necessários
	Processos padronizados para a contrarreferência
	Instrumentos/suportes relevantes para a segurança do processo
	Uso de tecnologias de informação e comunicação em saúde
Papel do enfermeiro na gestão de alta ambulatorial	Educação para a alta ambulatorial
	Coordenação da alta ambulatorial
Práticas colaborativas	Segurança para a promoção da continuidade do cuidado na Atenção Primária à Saúde
	Trabalho em equipe
	Protocolos conjuntos
Corresponsabilização da pessoa e/ou familiar para com o autocuidado	

Das categorias encontradas, é possível destacar que “Comunicação interpessoal e interprofissional”, “Fragilidade de conhecimento para uma adequada alta ambulatorial”, “Dificuldades para a contrarreferência da pessoa do ambulatório para a Atenção Primária à Saúde” e “Recursos para uma adequada contrarreferência” apresentam os grandes desafios vivenciados pela instituição e pela RAS para consolidação da continuidade do cuidado de pessoas com doenças crônicas.

*Eu acho que a comunicação é um dos principais fatores para que eu não consiga encaminhar esse paciente para a UBS.* (Profiss. R)

*Não sei se os processos são claros também, porque teria que preencher, eu nunca acesso. Como é que se faz uma contrarreferência? Já fiz algumas, mas tem que preencher algum documento, não sei onde. Eu não sei se esse processo é totalmente claro para nós, assim, eu acho.* (Profiss. N)

*Talvez se a gente tivesse um sistema integrado, né? Porque aqui a gente dá um papel pro paciente. Se esse papel vai chegar lá, físico, não sei.* [...] *e realmente os sistemas digitais eletrônicos não unificados, acho que é uma coisa que dificulta.* (Profiss. O)

Por sua vez, as categorias “Critérios para a alta ambulatorial”, “Papel do enfermeiro na gestão de alta ambulatorial”, “Práticas colaborativas” e “Corresponsabilização da pessoa e/ou familiar para com o autocuidado” evidenciam possíveis estratégias para o alcance desta continuidade.

*Na maioria das vezes, é assim: a gente avalia o paciente; vê quem são aqueles que têm doenças menos graves e que têm todas as medicações disponíveis e exames disponíveis para seguir na unidade de saúde; faz contrarreferência; e dá alta do ambulatório.* (Profiss. M)

[...] *a gente consegue fazer todo o esclarecimento do paciente novamente. Orientar de que ele vai para a unidade básica, que vai ser acompanhado, que isso está tudo estipulado e de que existe a possibilidade de o médico acompanhar* [...] *então, a gente explica tudo isso para ele nessa consulta de enfermagem.* (Profiss. W)

[...] *agora do que é a responsabilidade de cada um. Uma é a nossa, outra é da atenção básica, e o paciente tem a responsabilidade dele, com o tratamento dele.* [...] *a nossa terminou aqui; ali começa a da atenção básica; e aqui começa do paciente.* (Profiss. A)

Como resultado das oficinas, foi construído o fluxo do processo da gestão de alta ambulatorial, validado pelos participantes do estudo na última oficina. Esse processo ocorre em três momentos específicos: atendimento da especialidade; atendimento do enfermeiro ambulatorial; e registro da gestão de alta ambulatorial (Figura 1).

**Figura 1:** Fluxo do processo da gestão de alta ambulatorial, Curitiba, Paraná, Brasil

*GAA - gestão de alta ambulatorial; UBS - Unidade Básica de Saúde; SMS - Secretaria Municipal de Saúde.*

A análise de todos os dados coletados no estudo proporcionou à pesquisadora uma profunda compreensão da realidade estudada, além de subsidiar os fundamentos para a construção do modelo de gestão de alta ambulatorial^(24)^. A Figura 2 representa o modelo de gestão de alta ambulatorial idealizado.

**Figura 2:** Modelo de gestão de alta ambulatorial, Curitiba, Paraná, Brasil

A criação deste modelo posiciona a pessoa que necessita de cuidados de saúde no centro do processo, com foco em garantir sua transição adequada dos serviços ambulatoriais da atenção terciária para a Unidade Básica de Saúde de referência, promovendo a continuidade do cuidado. Destaca-se que os profissionais de saúde, tanto da atenção primária quanto da terciária, devem atuar junto à pessoa, e esta, por sua vez, deve ser agente ativo no processo de cuidado.

A comunicação é essencial para o funcionamento adequado do processo, tanto entre os profissionais, a pessoa e seus familiares quanto entre os próprios profissionais, dentro da instituição ou entre os diferentes níveis da RAS. A criação de uma interface eficaz de comunicação entre os níveis terciário e primário é fundamental, e depende de ferramentas regulatórias, meios de comunicação e acordos organizacionais, os quais, no modelo proposto, estão representados por uma linha pontilhada que conecta ambos os níveis. Essa linha é pontilhada porque simboliza o constante aprimoramento da comunicação, adaptando-se às mudanças tecnológicas e sociais, e ao desenvolvimento dos arranjos organizativos do SUS, especialmente no que diz respeito à regulação da RAS.

A designação do enfermeiro assistencial como elo central entre os níveis da RAS fortalece a continuidade do cuidado e destaca a importância desse profissional no atendimento ambulatorial. A colaboração entre todos os envolvidos é essencial para o alcance dos objetivos no processo de alta, tanto nas relações entre profissionais quanto entre a pessoa e sua rede de apoio, e entre os diferentes níveis da RAS. Contudo, essa colaboração depende, quase exclusivamente, do comprometimento dos profissionais, e cabe aos gestores incentivar e proporcionar um ambiente favorável ao desenvolvimento de práticas colaborativas ao longo de todo o processo.

O modelo proposto foi elaborado a partir da realidade de um hospital de ensino de porte terciário. Pode-se inferir que, para a sua implementação, o passo a passo a ser executado deve considerar as seguintes etapas:

- Definir o fluxo do paciente na RAS e na instituição, alinhamento da referência e da contrarreferência;
- Identificar nós críticos da instituição, perfil dos pacientes, número de pacientes vinculados, capacidade de atendimento ambulatorial e seu reflexo na dinâmica dos atendimentos de urgência e nas taxas de admissão/readmissão hospitalar;
- Definir critérios de alta ambulatorial, em que devem ser consideradas as condições do paciente para alta da atenção especializada e a capacidade de a atenção básica oferecer o suporte necessário para a continuidade do cuidado;
- Estabelecer um fluxo de comunicação efetivo entre equipe assistencial e regulação assistencial da instituição com as equipes da rede;
- Envolver o paciente e/ou familiares em todo o processo, desde a referência até a atenção especializada, esclarecendo os trajetos na rede de atenção, as idas e vindas, para que não se sintam abandonados no momento da contrarreferência;
- Estabelecer a padronização de ações com o gestor local, a fim de viabilizar o agendamento de consulta de retorno na atenção primária e conclusão do código de transação, conforme a pactuação com a instituição.

A realização de projeto piloto com uma especialidade com menor número de pacientes pode ser uma escolha aceitável para o alinhamento dos pontos frágeis no processo inicial.

Para que este modelo seja viável, em larga escala, ele precisa ser incorporado a políticas públicas que consolidem os processos assistenciais no SUS, respeitando os princípios de universalidade, integralidade e equidade. A operacionalização do modelo exige critérios claros de alta ambulatorial por especialidade, o protagonismo do enfermeiro no processo de gestão de altas, o comprometimento da pessoa com a própria saúde, a colaboração da rede para garantir o agendamento da consulta de retorno na Unidade Básica de Saúde pelo enfermeiro da atenção especializada, e o incentivo às práticas colaborativas em todos os níveis de atenção.

## DISCUSSÃO

A análise de conteúdo revelou aspectos importantes para elaboração do modelo idealizado. Entre eles, destaca-se a comunicação interprofissional e interpessoal, que emerge como um fator essencial para gestão da alta ambulatorial. Essa comunicação é fundamental tanto entre os profissionais de saúde e entre as pessoas quanto entre os próprios profissionais da instituição e entre os profissionais de diferentes níveis da RAS. Esse processo envolve a troca de informações relevantes para o cuidado da pessoa, considerando o contexto passado, presente e planos futuros. A deficiência na comunicação entre profissionais de saúde e pessoas/famílias pode comprometer a continuidade do cuidado^(3,8,25,26)^.

A eficiência na continuidade do cuidado está diretamente relacionada às interações entre os diferentes atores ao longo do percurso assistencial, à comunicação adequada e à abordagem interprofissional centrada na pessoa. Falhas na troca de informações interprofissionais podem resultar em duplicidade de atendimentos ou realização de exames desnecessários, enquanto falhas na orientação das pessoas em acompanhamento podem favorecer baixa adesão ao tratamento e, por conseguinte, uso inadequado de medicamentos e incompreensão do próprio papel no autocuidado^(4,25,27)^.

Outro aspecto relevante é a necessidade de que os profissionais envolvidos na alta ambulatorial estejam bem informados sobre os recursos da RAS e os processos de contrarreferência. Profissionais que dominam esses procedimentos conhecem os serviços disponíveis na rede e compreendem as necessidades individuais das pessoas, estando mais preparados para assegurar a transição do cuidado após a alta ambulatorial^(3)^.

A definição de critérios para a alta ambulatorial pode auxiliar na identificação de pessoas aptas para a alta, além de subsidiar os profissionais na construção de estratégias conjuntas com a APS para a continuidade do cuidado de pacientes com condições complexas. As dificuldades na contrarreferência da pessoa do ambulatório especializado para a APS demonstram a fragilidade da RAS, especialmente no que diz respeito ao acesso aos serviços de saúde. Facilitar esse acesso é essencial para promover a tão almejada continuidade, permitindo que as pessoas recebam atenção da condição de saúde conforme suas necessidades^(12)^. A barreira no acesso reforça a insegurança de pacientes e profissionais sobre a qualidade da APS, o que aumenta a resistência à alta ambulatorial^(2,3)^.

Para possibilitar uma contrarreferência eficaz, é fundamental contar com profissionais qualificados e comprometidos com a colaboração em rede. Estudos apontam que o enfermeiro de ligação desempenha papel fundamental na transição e continuidade do cuidado entre os serviços hospitalares e a assistência ambulatorial^(28-31)^. Ademais, processos bem estruturados e sistemas informatizados integrados são essenciais para otimizar a gestão da alta ambulatorial^(32-36)^.

O papel do enfermeiro na coordenação da alta ambulatorial é essencial, especialmente por meio de ações educativas. No modelo de gestão de alta ambulatorial proposto, busca-se restabelecer o vínculo entre os pacientes e a APS, no sentido de promover uma comunicação aberta e eficaz, o compartilhamento de informações relevantes, e o engajamento na tomada de decisão sobre o cuidado. Isso não se restringe à transmissão de informações clínicas, mas envolve o fortalecimento da relação de confiança e respeito mútuo entre todos os envolvidos^(35,36)^. A educação em saúde é uma prática essencial para viabilizar que pacientes e familiares compreendam seus tratamentos e saibam como acessar os serviços disponíveis na RAS. Esse processo educativo, no qual o enfermeiro desempenha papel central, é essencial para que os pacientes assumam um papel ativo no próprio cuidado^(35-37)^.

A adoção de práticas colaborativas entre os diversos atores da RAS contribuirá para a efetividade da continuidade do cuidado e consolidação dos princípios do SUS. Para isso, é imprescindível que os profissionais estejam comprometidos com a promoção da continuidade assistencial^(38)^. Além disso, é essencial estruturar e padronizar a troca de informações entre os serviços^(39,40)^.

A participação responsável da pessoa e/ou familiar é fator determinante para a continuidade do cuidado. Para que isso ocorra, é necessário que eles compreendam os serviços da RAS e saibam como acessá-los^(25)^. Dessa forma, uma abordagem integrada e centrada na pessoa, baseada na confiança, comunicação eficaz e colaboração interprofissional, é essencial para promover uma transição segura e um cuidado de qualidade ao longo do sistema de saúde.

### Limitações do estudo

As limitações envolvem a quantidade reduzida de pesquisas sobre o tema e a ausência da consolidação plena do modelo proposto, que ainda demanda investigações futuras para aferir seu impacto. O desafio reside na estruturação definitiva do modelo e na mobilização dos gestores para ampliá-lo, aspecto que será explorado em estudos posteriores.

### Contribuições para a área da enfermagem, saúde ou políticas públicas

O estudo contribui significativamente para o campo da enfermagem, ao evidenciar o papel central do enfermeiro na gestão do processo de alta ambulatorial e na continuidade do cuidado. Ademais, demonstra a necessidade de implementação de políticas públicas para a continuidade do cuidado e consolidação da APS como coordenadora da atenção à saúde no SUS.

## CONSIDERAÇÕES FINAIS

A presente pesquisa possibilitou a elaboração de uma proposta de modelo assistencial para gestão de alta ambulatorial, enfatizando a continuidade do cuidado e o papel do enfermeiro como coordenador desse processo. Evidenciou-se que a efetividade do modelo depende de comunicação clara e eficiente, do compartilhamento adequado de informações, e do reconhecimento do enfermeiro como articulador. Adicionalmente, a capacitação dos profissionais sobre os recursos disponíveis na rede, aliada à criação de protocolos bem definidos e construídos de forma integrada entre a atenção especializada e a APS, pode fortalecer a colaboração entre os diferentes níveis da RAS. No entanto, o sucesso dessa estratégia depende, fundamentalmente, do engajamento da pessoa como protagonista do seu próprio cuidado.

## Data Availability

Os dados de pesquisa só estão disponíveis mediante solicitação.
